# How good is the obesity associated with blood groups in a cohort of female university going students?

**DOI:** 10.12669/pjms.342.13633

**Published:** 2018

**Authors:** Shireen Jawed, Komal Atta, Saba Tariq, Farah Amir

**Affiliations:** 1 Dr. Shireen Jawed, MBBS, M.Phil. Associate Professor, Physiology Department, Aziz Fatima Medical College, Faisalabad, Pakistan; 2 Dr. Komal Atta, MBBS, M.Phil. Assistant Professor, Physiology/Sr.Coordinator School of Optometry, The University of Faisalabad, Faisalabad, Pakistan; 3 Dr. Saba Tariq, MBBS, M.Phil. Assistant Professor, Pharmacology and Therapeutics University Medical and Dental College, Faisalabad, Pakistan; 4 Dr. Farah Amir, MBBS, M.Phil. Professor, Physiology Department Aziz Fatima Medical College, Faisalabad, Pakistan

**Keywords:** Obesity, Blood groups, BMI, Stadiometer

## Abstract

**Objective:**

To find out frequency of obesity in female University students in Faisalabad and to investigate its association with blood groups of ABO system.

**Methods:**

A cross sectional study was conducted with a sample size of 200 female University students, recruited from the Faisalabad based institutes from May 2017 to July 2017. Relevant information was taken by administering questionnaire. Height in meters and weight in kg were taken by stadiometer. BMI was calculated using formula BMI=weight in kg/height m^2^. Blood groups were determined by classic (antigen-antibody agglutination test). The data was analyzed through SPSS 20. Descriptive were presented as mean± SD and association of BMI with blood groups was assessed by regression analysis. P value ≤0.05 deemed statistically significant.

**Results:**

Out of students, 192 attempted the questionnaire and participated in study (96% response rate), 30% of the 192 females were obese, distribution of ABO blood group showed 43%, followed by O, A and AB. 90% were Rh positive and 10% were Rh negative. Blood group O showed a trend towards obesity and blood group AB showed a trend towards lean body.

**Conclusion:**

The blood group O showed the significant positive association with obesity. Population with blood group O showed greatest susceptibility to be overweight and obese.

## INTRODUCTION

Obesity is prevalent globally in all age groups at an alarming rate. WHO states burden of obesity in urban areas of Pakistan from 22-40%.^1^

Finding the predisposing factors of obesity is a major concern for researchers due to its grave consequences like hypertension, hypercholesterolemia, diabetes mellitus type II, myocardial infarction, infertility, obstructive sleep apnoea,^1^ osteoarthritis and cancers. It also affects the cognition levels and performance. It has been associated with poor quality of life due to its psychosocial consequences and reduced life expectancy.^2^

It is an emerging epidemic in our country involving children as well as adults.^1^ Pakistan ranked 9^th^ out of 133 countries in the global burden of disease as mentioned in a report published in 2014.^3^ Due to the continuous rise in prevalence of obesity in Pakistan, it is essential to create awareness among the Pakistani population about harmful effects of obesity and identify the population at risk, so that it may be preventable in the future.

Genes for the ABO blood grouping system are located on chromosome 9q34. Evidence of correlation between polygenic modes of expression of hypertension, diabetes have been established with ABO genes in mice models, however the exact interlooping of genes is yet to be ascertained as a proper relation is difficult to assess in polygenic expression.^4^ Several researchers in the past have reported the linkage of obesity with ABO blood groups,^4^,^5^ while some have also disagreed to this relation.^2^,^6^ Evidences are also present suggesting the ABO system as a genotype marker for obesity^7^ but this relationship is still under intense investigation due to these conflicting results. New studies are needed to validate the association between blood groups and BMI, as the curiosity in this matter is unabated.

The present study was aimed to determine the frequency of obesity and its association with blood groups among university students. Poor eating habits, use of snacks and fast food consumption are leading to obesity in this population^8^ which can results in impairment of cognitive functions associated with poor academic performance and grades.^8^

## METHODS

This cross sectional study was performed among the undergraduate students of Faisalabad based institutes during May 2017 to July 2017. Students from various departments were involved in this study namely Department of Nutrition and Dietetics (DND), Department of Physiotherapy (DPT), Optometry and MBBS. A total of 200 students of age 17-25 years of the university were enrolled by convenience sampling. Sample size was calculated using the following formula:

Necessary sample size = (z-score)^2^ x Standard deviation x (Standard deviation-1)

(Margin of error)^2^

Where z-score at 95%=1.96,

Standard deviation= 0.5

Margin of error =7%

Relevant information including race, ethnicity, place of residence, age, past medical history and family history were collected by administering a structured questionnaire. Out of 200 only 192 properly attempted the questionnaire (96% response rate), rest were refused to participate in this study.

Study subjects were divided into groups on the basis of their blood group A, B, AB and O. Informed written consent containing the objective of study was taken from the participants and complete confidentiality was assured. Stadiometer was employed to check bodyweight in kilograms, with subjects not wearing shoes and in light clothing. Height was measured by the same instrument to the nearest 0.1cm. BMI was calculated by the formula BMI=weight in kg/height in m^2^. BMI ≥ 25kg/m^2^ was considered as obesity as per WHO guidelines for Asian populations.^9^ Cut of points of BMI to determine obesity in Asians are lower than age and sex matched Europeans due to presences of greater body fat percentages in them.^10^

ABO typing was done by classic (antigen-antibody agglutination test) method of making slides. Aseptic measures were ensured and blood was taken by finger pricking with sterile lancet. Three clean slides were labeled as “A”, “B” and “D” followed by placing drops of blood over them. Anti-sera A, antisera-B and antisera D were added on each slide and mixed with blood properly. The agglutination reaction was used to check blood groups.^11^

### Statistical analysis

Statistical analysis was conducted on SPSS (Statistical Package for Social Software) version 20. Demographic data of the study population was evaluated by descriptive statistics. Continuous variables like age and BMI were expressed as mean ± standard deviation. Differences in means of demographics and variables between study groups were analyzed using Analysis Of Variance (ANOVA). Linear regression was applied to see the association between blood groups and BMI. P value ≤ than 0.05 was considered significant.

## RESULTS

The various anthropometric parameters were compared amongst the different blood groups and are mentioned in Table-I. Of these age and BMI were found to be statistically significant. Out of the total population, 29.7% was found to be obese.

**Table-I:** Comparison of anthropometric measurement between study groups.

Study groups (n=192)	Age (years) Mean ± SD	Height (meters) Mean ± SD	Weight (kg) Mean ± SD	BMI (kg/m^2^) Mean ± SD
A [n=47(16.2%)]	19.8 ± 2.18	1.56 ± 0.316	59.3 ±14.3	23.6±5.28
B [n=82(28.2%)]	19.2 ±1.65	1.60± 0.64	56.1±11.25	22.1±5.25
AB [n=10 (3.4%)]	18.2±0.632	1.63±0.58	53.1±5.61	19.53±2.35
O [n=53(18.2%)]	19.3±1.56	1.93 ±2.23	64.6±21.9	25.5±10.2
P value	0.049*	0.518	0.145	0.037*

ANOVA for comparisons of means, Statistically Significant value at P ≤ 0.05, BMI= Body mass index.

Distribution of ABO blood groups is indicated in Fig.2, highest percentage of study population were blood group B (42.7%) followed by O, A and AB respectively (Fig.I).

**Fig.1:**
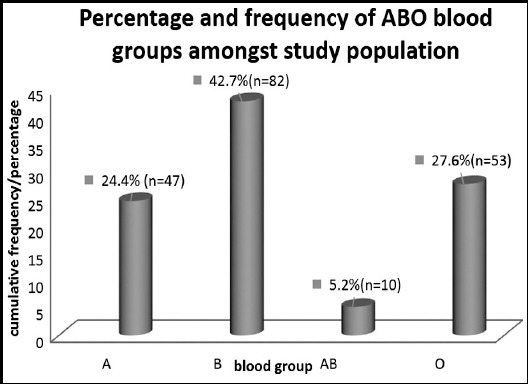
Frequency and percentage of ABO blood groups in study population.

Frequency and percentage of Rhesus blood groups were showing the predominance of positive rhesus groups in the study population174 (90.6%), while only 18 (9.4%) of population was rhesus negative. Highest BMI was recorded in blood group O with mean ±standard deviation of 25.5±10.2 and lowest was recorded in AB blood group with mean ±standard deviation of 19.53±2.35. (Table-I).

Rresults of Linear regression for relationship between blood groups and BMI are presented in Table-II, showing negative association between blood group A and BMI (β= -0.66) indicating that a greater BMI was associated with less people of blood group A. The regression was significant positive in blood group O ((β= 2.53, P=0.025 *) indicating that blood group O had a direct relationship to raised BMI.

**Table-II:** Linear regression between bloods groups and BMI.

Blood groups	BETA	P Value	95% Confidence Interval
A	- 0.066	0.844	0.724 - 0.592
B	1.662	0.10	0.36 - 3.68
AB	5.51	0.29	- 4.89 - 5.926
O	2.53	0.025*	0.327 – 4.751

**Table-III:** Percent of lean, overweight and obese subjects in each blood group.

Study groups (n= 192)	Underweight (BMI<18.5 kg/m^2^)	Normal weight (BMI=18.5-22.9 kg/m^2^)	Overweight (BMI=23-24.9 kg/m^2^)	Obese (BMI ≥ 25 kg/m^2^)

	Frequency (%)	Frequency (%)	Frequency (%)	Frequency (%)
Blood group A [n=47(16.2%)]	8 (20.5)	14(35.9)	3(7.7)	14 (35.9)
Blood group B [n=82 (28.2%)]	17(20.7)	34(41.5)	9(11)	22(26.8)
Blood group AB [n=10(3.4%)]	4(40)	5(50)	1(10)	0
Blood group O [n=53 (18.2%)	4(6)	20(32)	12(19)	25(41)

Percentages are compared by Pearson Chi-Square. P value =0.036* Statistically Significant value at P ≤ 0.05 Classification of BMI is taken as per criteria of WHO for Asians.

## DISCUSSION

The primary aim of the study was to determine the frequency of obesity among the young girls. Increasing its prevalence is attributed to common trends of consumption of snacks, energy drinks, poor eating habits, lack of physical activities^6^, spending much of their time on computers and TV watching among the young individuals.^8^ Lack of health awareness is also contributing factor. There are well established supporting evidences regarding the harmful consequences of obesity which are available in past documentation of various researches.^1^,^2^,^6^ Similarly new researches are required for the screening of the youth who are vulnerable for obesity and its adverse effects.

Many previous studies had reported the great importance of ABO blood group system in determining disease and health states. Not only ABO blood group system is important for determining hypertension, erythroblastosis fetalis, blood transfusion and exchange reactions^12^ but has also been shown to be relevant to conditions such as osteodysplasia and as genetic marker of obesity.^12^ More recent developments are exploring the relationship between ABO blood grouping and obesity^13^, which was also one of the main objectives of current study. Relationship between Lewis blood grouping and obesity was extensively studied by Hein HO and his colleagues^13^ and many other researchers; however the association between ABO blood group system and obesity is still to be hypothesized due to conflicting results documented by various researchers. With regards to susceptibility of various blood groups with obesity, our study demonstrated individuals with blood group O to be more prone to being overweight and obese. This was in congruent with many previous studies conducted to see the correlation between blood groups and obesity carried worldwide and locally also.^14^ present study population was 29.7% obese out of which blood group O showed greatest susceptibility to be overweight. This was in consistence with results from India, which also demonstrated blood group O to be most significantly related to obesity.^14^ Egyptian population also demonstrated blood group O to be strongly linked with increased BMI,^15^ supporting the results of current study, this was however in contrast to studies from India by Chandra T et al.^12^ and Africa which showed B to be the most common blood group associated with obesity,^12^,^16^ their finding is most probably because of presence of more lipid contents in blood group B and A as compared to blood group O,^17^ but this fact is still required to be studied.

Our study showed that the most common blood group in our study population was B, followed by O, A and AB. These results were similar to those reported by studies conducted in Swat, District Nowshera, Lahore, Gujranwala, Multan, Faisalabad and many other cities of Punjab.^14^,^18^-^20^ Incompatible to our results, studies from northern area reported blood group A to be the commonest blood group.^21^ Considering the least common blood group, all aforementioned studies were consistent with our study demonstrated blood group AB to be the least commonest blood group.^14^,^18^,^22^ It was also consistently shown that like in our results, all ethnicities demonstrated greater population of Rhesus Positive individuals as compared to Rhesus negative. Confirmatory studies are required to elucidate the relationship between obesity and blood groups and to delineate the mechanistic basis of this association. There is also need to identify the young peoples at risk of obesity in various regions.

Health awareness programs should be arranged for our adolescent population in schools and colleges to educate them about the grave effects of obesity and its contributing factors like lack of balance diet, consumption of junk food, TV viewing, gaming and lack of exercise. Health awareness through media and use of posters at major public places and institutes may be helpful. Young adults should be encouraged for balanced nutrition and physical activity to avoid trends of weight gain to protect them from health issues and for their better cognitive functions and performance in future.

### Limitations

The limitations of current study included a single gender (females), small sample size as well as lack of data on Rhesus negative blood groups owing to the rarity of these donors.

## CONCLUSION

Obesity is most commonly prevalent among the individuals with blood group O. Further studies need to be carried out to investigate relation between BMI and blood group O more thoroughly by investigating the mechanism behind it, to come up with more definitive conclusion.

### Author’s Contribution

***SJ:*** Study design, data collection, statistical analysis, interpretation and of results, and formulation of tables, manuscript writing and revising it critically for important intellectual content

***KA:*** Study design data collection, Statistical analysis, writing the manuscript. Formulation of tables.

***ST:*** Study design Acquisition of data, interpretation of results, editing and formatting the manuscript. Final approval of the manuscript

***FA:*** Data collection, Statistical analysis, interpretation of results, Final approval of the manuscript.
